# Fc-Binding Ligands of Immunoglobulin G: An Overview of High Affinity Proteins and Peptides

**DOI:** 10.3390/ma9120994

**Published:** 2016-12-08

**Authors:** Weonu Choe, Trishaladevi A. Durgannavar, Sang J. Chung

**Affiliations:** 1 Department of Chemistry, Seoul National University, Seoul 151-742, Korea; chweha@gmail.com; 2 Research Institute of Biomolecular Chemistry, Dongguk University, Seoul 100-715, Korea; trishalad@gmail.com

**Keywords:** Fc-binding peptide, antibody, targeted drug delivery, affinity column chromatography, Protein A, Protein A mimics, Protein G, Protein L

## Abstract

The rapidly increasing application of antibodies has inspired the development of several novel methods to isolate and target antibodies using smart biomaterials that mimic the binding of Fc-receptors to antibodies. The Fc-binding domain of antibodies is the primary binding site for e.g., effector proteins and secondary antibodies, whereas antigens bind to the Fab region. Protein A, G, and L, surface proteins expressed by pathogenic bacteria, are well known to bind immunoglobulin and have been widely exploited in antibody purification strategies. Several difficulties are encountered when bacterial proteins are used in antibody research and application. One of the major obstacles hampering the use of bacterial proteins is sample contamination with trace amounts of these proteins, which can invoke an immune response in the host. Many research groups actively develop synthetic ligands that are able to selectively and strongly bind to antibodies. Among the reported ligands, peptides that bind to the Fc-domain of antibodies are attractive tools in antibody research. Besides their use as high affinity ligands in antibody purification chromatography, Fc-binding peptides are applied e.g., to localize antibodies on nanomaterials and to increase the half-life of proteins in serum. In this review, recent developments of Fc-binding peptides are presented and their binding characteristics and diverse applications are discussed.

## 1. Introduction

Recent decades have seen the development of a myriad of applications of antibodies (Abs) and related proteins, and have become one of rapid growing classes of protein pharmaceuticals [1,2,3,4,5,6]. Abs are members of the immunoglobulin (Ig) super family and amount up to 20% of the plasma proteins. As part of the humoral immune response, Bursa lymphocytes (B-cells) are activated to differentiate into plasma cells that produce antibodies to combat pathogens and foreign substances in the host. All Abs share a common structure where two identical heavy chains glycoproteins and two identical poly peptide light chains are joined by disulfide bonds to form a large Y-shaped protein. These macromolecular proteins of ≥150 kDa have two fragment antigen binding (Fab) domains where their cognate antigens bind with high selectivity and specificity. In addition, Igs display two fragment crystallizable (Fc) regions that are the primary recognition site for e.g., cell surface receptors of effector cells, immune proteins, and other antibodies. Among the five different classes of antibodies, IgGs are the most abundant Ab and are also the second longest circulating protein (up to 21 days, except for IgG3 which is ~7 days) in the blood stream (albumin being the longest). The long half-life of IgG is attributed to the neonatal Fc-receptor (FcRn)-mediated endosomal salvation thereof (IgG3 lacks a crucial histidine residue, His435, involved in FcRn binding) [7,8,9]. In pharmaceutical applications of antibodies, IgG (and its subclasses) is the most prevalent Ab used. For instance, IgGs are applied as integral parts of therapeutics against a wide range of diseases (e.g., inflammation, cancer, autoimmune, and infectious diseases) and are also crucial in a variety of immunoassays such as ELISA and Western blot analysis. The wide-spread use of IgGs has necessitated the development of methods to manufacture, isolate and detect specific antibodies from complex sample mixtures [10]. In general, the production of Abs involves their expression in recombinant mammalian cell cultures followed by diverse purification steps, e.g., centrifugation, filtration, and chromatography [11]. 

Traditional, Ig-binding proteins, generally produced by gram-positive bacteria, have been widely explored in Ab isolation procedures [12,13,14]. In particular, Protein A (SpA), a virulence factor produced by *Staphylococcus aureus*, has been used in the chromatographic purification of Abs due to its high affinity towards IgGs from different species [15]. When the purity requirements of the final Ab is very strict, the utilization of these bacterial proteins in Ab purification is limited [16]. Besides the high production cost, one of the major problems encountered when using bacterial proteins in affinity purification of Abs is the contamination of the purified Ab samples with traces of these proteins (better known as ligand leakage). The contaminants may invoke an undesirable immune response when the Ab is used in the human body. To expand the pool of Ab binding ligands and to overcome the drawbacks associated with the use of bacterial proteins, recent decades have witnessed a great effort in the development of new Ab binding ligands [17,18]. Based on the application of the Ab affinity ligands, the orientation of the bound Ab is one of the major factors that should be considered. For instance, if the affinity ligand is used to bind Abs for usage in therapeutics and immunoassays, it is crucial that the Fab region of the Ab is not disturbed in order to enable binding to its cognate antigen. On the other hand, when the affinity ligand is intended for use in Ab purification, the binding site to the Ab is not of great importance as is the binding affinity.

Novel small molecules have been developed to mimic the binding properties of bacterial proteins to Abs and are extensively reviewed in literature [17,18,19]. Short peptides are known to selectively and strongly bind to a great variety of biomolecules. Especially cyclic peptides, as opposed to linear peptides, are reported to possess enhanced specificity and avidity towards their targets, in addition to superior enzymatic stability and conformation rigidity [20]. The straightforward synthesis, both synthetic and recombinant, allows for the generation of vast peptide libraries, and in combination with high throughput screening offers a powerful strategy to obtain high affinity binders for any given target. In particular, phage display is a robust technique that facilitates the identification of potent and selective peptide ligands to target Ab [21,22]. Our group actively develops applications of Fc-binding peptides and in this review we aim to give a brief overview of the traditional (bacterial protein) Fc-binding ligands and describe the development of short Fc-IgG binding synthetic peptides. Secondly, modern applications of small IgG binding ligands that target the Fc-domain of Igs will be described.

## 2. Immunoglobulin Binding Proteins

**Figure 1:**
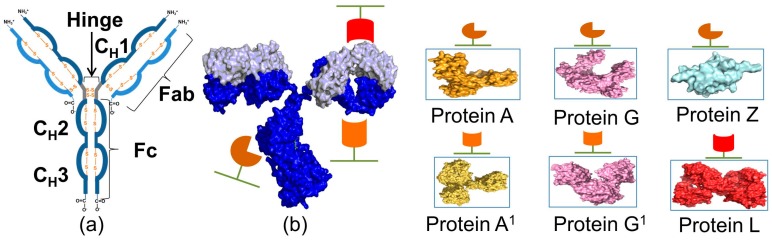
Illustration of an antibody and the most common bacterial proteins used. (**a**) Schematic representation of the different parts of an antibody; (**b**) pymol-generated crystal structure of IgG (pdb id: 1IGT), Protein A (pdb id: 1FC2), Protein G (pdb id: 1FCC), Protein Z (pdb id: 1Q2N), Protein A^1^ (pdb id: 1DEE), Protein G^1^ (pdb id: 1QKZ), and Protein L (pdb id: 1HEZ).

A variety of bacterial proteins are known to bind mammalian Igs, including Protein A, G, L, Z, and recombinant (fusion proteins) derivatives thereof (Figure 1, Table 1) [23,24,25,26,27,28,29]. SpA is known to specifically bind to the Fc-domain of Igs and is utilized in, e.g., in immunoprecipitation techniques, double sandwich immunoassays, and in affinity purification of Abs. The strong binding of SpA to various mammalian Abs (*K*_d_ ~ 10 nM to IgG1) is attributed to its five homologues binding domains (A–E) that targets the Fc-domain (between the C_H_2 and C_H_3 region) [12,30]. In addition, SpA also binds to the human Fab-heavy chain of the V_H_3 family [31]. The binding affinity of SpA varies for the different IgG subclasses, whereas IgG1, IgG2, and IgG4 show strong SpA binding, relative weak binding of SpA to IgG3 is observed [8]. 

Whereas SpA in general is preferred for rabbit, pig, dog and cats, Protein G (from Streptococcal origin) possess a better binding capacity for a broader range of mouse and human IgG subclasses (*K*_a_ 6.7 × 10^9^ M^−1^ towards human IgG) [32]. However, Protein G also has an albumin binding site which is one of the major constituents of serum proteins. Therefore, for Ab purification a recombinant Protein G that lacks the albumin binding site is preferred. Another bacterial Ig-binding protein is Protein L from Peptostreptococcus magnus. Protein L does not bind to the Fc-domain, but rather to the κ-light chain of IgG (*K*_a_ 5.7 × 10^7^ M^−1^) and can be used to isolate Ab fragments that do not contain the Fc-domain [23]. The great importance of Ig-binding proteins has stimulated research directed to improve their application as high affinity ligands (e.g., by mutations and synthetic derivatizations of the Ig-binding domain) [33]. In this vein, Protein Z, a synthetic protein modeled on the B-domain of SpA was reported [26,34]. In another approach, Braisted et al. designed a two-helix derivative of the Z-domain of SpA. Through the use of structure-based design and phage display optimization, a 33-mer peptide (Z33) was developed that binds to IgG1 with a *K*_d_ of 43 nM [35].

To improve the binding characteristics of the bacterial proteins, several fusion proteins were developed. In general, these fusion proteins combine the Ig-binding domains of the three main bacterial Proteins A, G, and L. Some interesting properties of these (fusion) proteins are listed in Table 1. In addition to fusion proteins, artificial affinity proteins of Ig are attractive alternatives to bacterial (fusion) proteins. However, the development of artificial Fc-binding proteins is beyond the scope of this review and for further information on this subject, the reader is referred to a recent review [36]. 

One of the drawbacks of Fc-binding bacterial proteins for use in affinity column chromatographic purification strategies of Ab is the relatively acidic elution condition. Under these harsh elution conditions, some Ab may aggregate or denature. Therefore, engineering of the pH sensitivity of bacterial Fc-binding protein has received much attention. One general applied strategy involves the systematical introduction of histidine residues to induce electrostatic repulsion between the affinity protein and the Ab under relative mild acidic conditions. The optimization of the pH response affinity using histidine-mediated electrostatic repulsion between Protein G and the Fc-domain of IgG was described by Watanabe et al. [37]. Tsukamoto et al. reported the engineering of SpA variants by a histidine-scanning library that allowed elution of Abs at a higher pH (∆pH ≥ 2) and bound IgG was eluted for 90% at pH = 5 [38]. Using site directed mutagenesis, Gulich et al. described the preparation of “destabilized” Protein Z mutants which allowed the elution of bound Abs at pH = 4.5, albeit that the affinity to IgG was slightly lower with respect to Protein Z [39]. Strauch et al. reported the computer design of a pH-sensitive IgG binding protein which has a *K*_d_ of ~4 nM at slightly basic pH (8.2) [40]. In contrast, at acidic pH (5.5) the binding to IgG is about 500-fold weaker. Two different strategies, i.e., histidine scanning and random mutagenesis, were employed to gain a mutant that has a higher binding affinity at pH = 7.4 than at pH = 4.5. Gera et al. described a pH sensitive Fc-IgG binding protein derived from the hyperthermophilic Sso7d scaffold [41,42].

In general, the orientation of Abs can drastically influence antigen binding hence site-directed immobilization may be crucial. Bacterial proteins that bind to Abs with high affinity in a site specific manner can be used for the grafting of Ab on e.g., solid surfaces or to covalently attach cytotoxic compounds [43]. To generate a covalent linkage between the Ab and the affinity ligand, typically incubation with a bifunctional reagent (a cross-linker) is used, e.g., dimethyl pimelimidate. Jung et al. reported the modification of Protein G with a benzophenone moiety (photo cross-linker) [44]. In a similar approach, the Z domain of SpA was constructed using a benzophenone derived artificial amino acid and was applied in the covalent labeling of Igs [26].

## 3. Immunoglobulin Binding Peptides and Peptidomimetics

**Figure 2:**
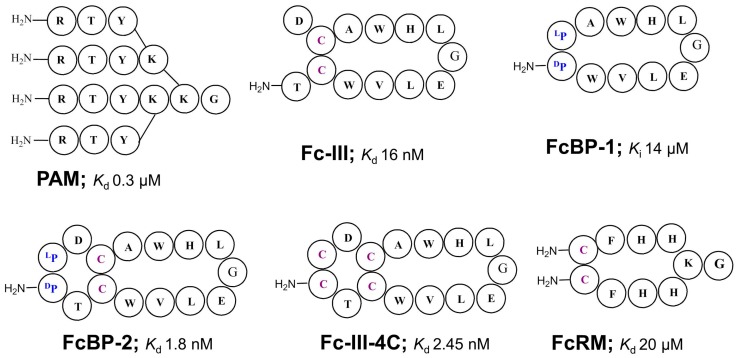
Structures of branched- and cyclic peptide-based high affinity ligands that bind to IgGs through interaction with the Fc-domain. The reported affinity constants are towards IgG. Elution pH of antibody from affinity ligand column: PAM, 3 or 9; Fc-III, 3.5; Fc-III-4C, 3.5; FcRM, 2.7 [42,45,50,51,54].

Currently, a vast number of short (cyclic) peptides are reported that can specifically bind to Ab and a representable selection of these peptides are depicted in Table 2 and the structures of branched and cyclic peptides are shown in Figure 2. 

In an approach to discover SpA mimetic affinity ligands, Fassina et al. (1996) screened a combinatorial peptide library composed from randomized tetramers [45]. After three rounds of selection, a dendrimeric peptide ligand with the formula (Arg-Thr-Tyr)_4_-Lys_2_-Lys-Gly, denoted as PAM (Figure 2) was identified as the most active ligand to inhibit binding of SpA to biotinylated IgG. Their findings indicated that PAM binds to the Fc-region of IgG with a *K*_d_ of about 0.3 μM and could target a broader range of IgG (from different species) compared to SpA. The apparent affinity constant between PAM and rabbit IgG was reported to be 3 × 10^5^ M^−1^. The PAM ligand was immobilized on column material and used in a one-step Ab purification from crude sera. The binding selectivity of immobilized PAM was found to be similar to SpA, and was found to be stable to column sanitizing conditions such as ethanol and 0.1 M sodium hydroxide. In addition, the prepared affinity column was used for up to 40 consecutive Ab purification runs without noticeable decrease of the column capacity. A later report from the same group dealt with the preparation of the all d-amino acids version of this ligand, called d-PAM, to suppress proteolysis thereof [46]. 

Using Emphaze™ as the solid support to immobilize d-PAM, Ab purification was reported with a recovery yield in the range of 60% to 90% and with a purity >90%. Furthermore, prolonged incubation with mouse serum showed excellent resistance to proteolytic degradation of the d-PAM column. Further structural refinement of d-PAM, i.e., the acetylation of the four N-terminal arginines with phenylacetic acid, gave d-PAM-Ф having improved binding characteristics [47]. The binding capacity of the solid-support immobilized d-PAM-Ф ligand was about one order of magnitude higher about 10 mg/mL with respect to d-PAM, giving IgG with a purity >90%. Elution of the bound Abs from the d-PAM-Ф column was achieved using 0.1 M acetate buffer (pH = 4). 

From a phage display decapeptide library (~4 × 10^8^ different peptides), Krook et al. reported the identification of TWKTSRISIF which has a high affinity for the Fc-domain of human IgG [48]. Phage display involves the fusion of randomized amino acid sequences to filamentous phage envelope proteins by genetic engineering of the phage genome. Phage display library allows the simultaneous screening of a gigantic number of peptides to identify high affinity ligands towards a specific target. In the reported approach, a preselection of the library was preformed using streptavidin-coated paramagnetic beads to eliminate phage clones that interact with streptavidin or with the plastic. Next, the reduced library was subjected to five rounds of selection against biotinylated IgG. After the incubation time, streptavidin-coated paramagnetic beads were used to isolate the Ab and the bound peptides were displaced using either SpA or low pH (2.2).

**Figure 3:**
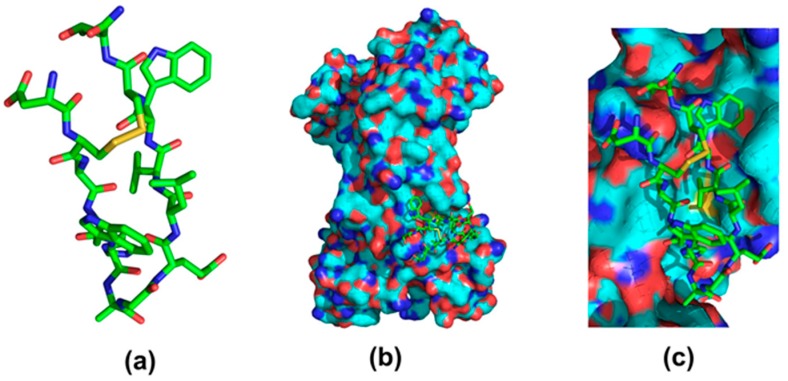
Structure of Fc-III binding to the Fc-region of IgG. (**a**) Fc-III (DCAWHLGELVWCT-NH_2_); (**b**) Full view; (**c**) close view. Pymol generated image from a PDB file (1DN2).

Around two decades ago (2000), DeLano and coworkers reported the identification of a small cyclic peptide that almost exclusively bind to the Fc-region of IgG [42]. In their study, the consensus binding site on the Fc-region of IgG was probed using a bacteriophage display peptide library of 4 × 10^9^ different peptides containing an intermolecular disulfide bond [22]. After several rounds of in vitro selections, a 13-mer Fc binding peptide (Fc-III, DCAWHLGELVWCT-NH_2_, Figure 3a) with an unusual high binding affinity towards the Fc-region of IgG was identified. Competitive assays of Fc-III with SpA showed that the binding of SpA to the Fc-domain was inhibited with a *K*_i_ of 25 nM. Albeit that Fc-III is much smaller in size with respect to SpA and Protein G (*K*_d_ about 10 nM), its binding to the Fc-region is only about a twofold weaker. To investigate the region and nature of the binding of Fc-III to the Fc-binding domain, the X-ray crystal structure of the Fc-III/IgG-Fc complex was determined at 2.7 Å (Figure 3b,c). The binding site on the Fc-domain of IgG is reported to be adaptive, exposed, nonpolar, and energetically important and primed for the interaction with multiple different molecules. Fc-III was found to be constrained in a *β*-hairpin conformation which is unrelated to known Fc-binding motifs, and despite having no structural similarities to natural Fc-binding proteins, Fc-III binds to the common binding site on the Fc-domain of IgG (the hinge region of the IgG-Fc domain). The importance of the internal disulfide bond was demonstrated by Dias et al. who found that reduction thereof in Fc-III completely diminished the inhibition of SpA towards Fc-binding (Table 3) [50]. 

The potential of Fc-III was demonstrated with the development of a re-useable (up to 60 runs) affinity purification column that gives high purity Ab (95%) [49]. Immobilization of Fc-III on sepharose had no significant effect on the binding affinity of Fc-III towards IgG and allowed elution of the bound Ab at pH comparable to Protein G affinity columns (pH = 3.2). Interestingly, it was found that the reduced Fc-III was able to bind to IgG and allow elution at less acidic pH (4.8). This is in contrast to the finding of Dias et al. who reported that reduction of the disulfide bond in Fc-III results in total loss of SpA inhibition towards IgG binding [50]. One hypothesis is that reduced Fc-III binds to a different region on IgG or it may that the disulfide bond is reformed upon exposure to air. 

**Figure 4:**
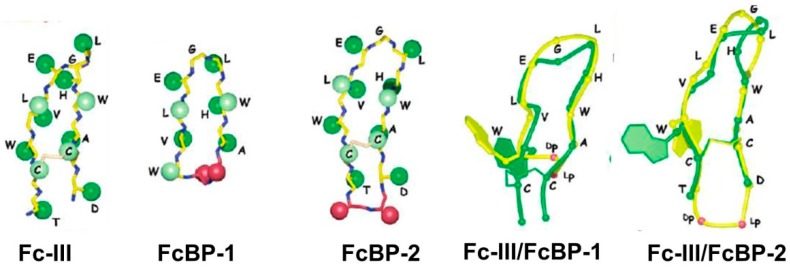
Superimposition of the backbone atoms of the bound structure of Fc-III (green). Adapted from [50], with permission from © 2006 ACS publications.

Interestingly, Dias and coworkers reported in 2006 a drastic improvement of the original Fc-III peptide towards Fc binding using a small backbone cyclic peptidomimetic [50]. In the construction of the so called protein epitope mimetic, a d-Pro-l-Pro template was used to induce and stabilize the β-hairpin conformation that is present in Fc-III [68]. Computer mimetic design modeled on Fc-III and the d-Pro-l-Pro template afforded two mimetics, FcBP-1 and FcBP-2 (Figure 4). NMR spectroscopic conformational analysis (ROEs and ROESY) of FcBP-1, which lacks the crucial disulfide bridge, revealed a well-defined backbone conformation and the presence of a tight turn, however, the characteristics of the computer predicted *β*-hairpin was not observed. In contrast, FcBP-2, that comprises the d-Pro-l-Pro template in addition to the critical disulfide bridge, showed NOE correlations that are consistent with the expected *β*-hairpin backbone. Whereas FcBP-1 did not show significant inhibition of SpA binding to human IgG1, FcBP-2 was found to have an 80-fold higher affinity for the Fc-domain of human IgG1 with respect to Fc-III (*K*_i_ of 0.4 nM).

Gong et al. reported a bicyclic derivative of Fc-III, based on the findings of Dias et al. (described above) detailing the enhanced binding affinity of the double cyclic FcBP-2 [51]. It was proposed that the incorporation of the d-Pro-l-Pro in addition to the N to C termini cyclization to construct FcBP-2 would present various difficulties in the recombinant production thereof. Therefore, two additional cysteine residues were added instead of the d-Pro-l-Pro template to the N- and C-termini of Fc-III, respectively, resulting in the formation of two internal disulfide bridges. The bicyclic derivative, Fc-III-4C, showed a 30-fold increased binding affinity towards human IgG, over Fc-III and similar to FcBP-2. Furthermore, Fc-III-4C was found to bind various IgGs from different species with *K*_d_ values in the low nanomolar range.

Interestingly, Camperi et al. reported in 2003 the affinity purification of Ab using a small tetrapeptide, APAR, that was identified from a combinatorial synthetic library [53]. Immobilized APAR on agarose showed a maximum Ab binding capacity of 9.1 mg/mL and gave electrophoretically pure anti-Granulocyte macrophage-colony stimulating factor monoclonal Ab (produced in mouse) in a yield of 95%. Verdoliva et al. described the disulfide bridged synthetic peptide (NH_2_-Cys-Phe-His-His)_2_-Lys-Gly-OH, denoted as FcRM, as a ligand for IgG [54]. The dissociation constant of FcRM was reported to be 20 μM and FcRM proved useful as an affinity ligand in the purification of IgG. Surprisingly, FcRM was shown to bind Ab fragments containing both Fab- and Fc-domains from different Ab isotypes which indicate that FcRM targets at least two different binding sites on the Ab. 

About 10 years ago, Yang and coworkers reported a linear hexa-peptide that is able to bind to the Fc-region of IgG [55,56]. The screening of a synthetic library of hexa-peptides composed of N-terminal histidine followed by aromatic amino acids performed. After a three-step screening process, three candidates were identified and among them the hexamer HWRGWV was found to be suitable for chromatographic purification of human IgG from cell culture medium. The purity of the Ab obtained with this ligand was comparable to commercially available resins, and slightly milder elution conditions (pH = 4) could be used with respect to the use of SpA (pH = 3.5). The hexameric peptide was found to bind to all the subclasses of human IgGs, and furthermore, displays non-specific interactions towards human IgM and to a lesser extend to human IgA. HWRGWV binds to the Fc-region of Igs, however, it does not compete with SpA or Protein G [69]. The hexamer immobilized on Toyopearl AF Amino 650M resins has an Ab binding capacity of 28 mg/mL and was used to isolate human IgG in a yield as well as purity of 95% and the affinity column could be used up to 35 purification cycles. In 2013, the same group reported the preparation of an mRNA-display library of cyclic pentapeptides and evaluated their Fc-binding properties [60]. Solid-phase cyclisation of the mRNA-fusion peptides was achieved in good yield (87%) and purity (94%). The most promising candidate from the display library, cyclo[*Link*-M-WFRHY-K] was synthesized directly on the chromatographic Toyopearl resin and used for the isolation of human IgG. The dissociation constant of cyclo[*Link*-M-WFRHY-K]-Toyopearl was found to be 7.6 μM and have a total Ab binding capacity of 20 mg/mL. The purification of monoclonal IgG from industrial CHO cell culture supernatants was reported with a yield of ~96% and a purity of ~93%. Recently, the modification of the above described hexamers with unnatural amino acids was reported to gain resistance to proteases [57]. 

Sugita and coworkers have reported two high affinity octameric peptides towards the Fc-region of IgG [61]. In their report, peptides derived from the outer membrane amino acid sequence of the IgG Fcγ receptor, one of the well-known IgG Fc-binding proteins, were screened. Utilizing a spot-synthesized peptide array, the octameric peptides NKFRGKYK and NARKFYKG were found to have high binding affinity for the Fc-region of IgG (both mouse and human), with association constants of 8.9 × 10^6^ and 6.4 × 10^6^ M^−1^, respectively. Both peptides were used in affinity chromatographic purification of Abs in yields of 69% (NKFRGKYK) and 80% (NARKFYKG) with purities of 83% and 68%, respectively. The recovery of the Ab form the resin is pH dependent, elution at pH = 6 give low recovery yield whereas the highest recovery yield was reported when elution is performed at pH = 4. 

Interestingly, Tsai and coworkers reported in 2014 a strategy to identify small peptide ligands that bind to the Fc-region of human prostate specific Ab derived from mouse IgG2a [66]. In their approach, molecular docking studies using naphthalene and end-capped tryptophan were used followed by molecular dynamics simulations to find the most suitable ligand. After additional optimizations, the tetrameric peptide RRGW was found to have a high binding affinity towards the bottom of the Fc-region of Ab with a *K*_d_ of 5.6 × 10^−10^ M. SPR analysis was used to determine the recognition efficiency and orientation factor of antibodies towards RRGW bound surface. SPR studies indicated high binding affinity (*K*_a_ = 1.8 × 10^9^ M^−^^1^) of RRGW and mouse IgG2a. Using secondary Ab, the orientation factor of IgG2a adsorption on to surface bound RRGW was examined and was found to be much higher compared to other negatively and positively charged surfaces. 

For the binding of Ab on the surface of a quartz crystal microbalance biosensors, Yoo et al. reported the identification of a heptameric peptide KHRFNKD (ABP1) with a high and specific affinity to rabbit IgG [67]. A phage-displayed heptameric peptide library was screened for peptides that specifically bind to the Fc-domain of rabbit IgG. After four rounds of biopanning, the most active peptides were synthesized and analyzed and ABP1 was found to have a *K*_d_ of 2 × 10^−^^8^ M towards rabbit IgG, however weak binding to bovine, goat, human, and murine IgG was observed. The ABP1 fabricated biosensor was used to capture Abs without the loss of activity. 

Following a biomimetic design strategy based on SpA, Zhao et al. described in 2014 the identification of the octapeptide FYWHCLDE with a dissociation constant of 1.5 μM [62]. In their strategy, based on the SpA affinity motif, a 2173 member peptide library was build and evaluated using semi-flexible docking (Autodock Vina and RMSD) software. After the initial library was reduced to about 150 candidates, further computer aided modeling further reduced the library to 14 peptides. The most promising octameric peptide (FYWHCLDE) together with a negative peptide (FYTHCAKE) were selected for experimental verification. Immobilization of FYWHCLDE on Sepharose gel (with optimized ligand density of 31 μmol/mL) gave a maximal Ab binding capacity of 176 mg/mL with a binding affinity *K*_d_ of 2.4–3.7 μM [63]. The affinity column was reused up to 20 times showing a slight decrease (8%) of the original binding capacity. In the same year, two more octameric Fc-binding peptides (i.e., FYCHWALE, FYCHTIDE) were reported by Zhao et al., albeit that FYWHCLDE remained the most active peptide [64]. Interestingly, an antibody affinity column was described which employs FYWHCLDE and FYCHTIDE in a molar ratio of 2:1 [65]. The dual affinity ligand column showed synergistically enhanced binding affinity towards human IgG (*K*_d_ = 0.7 μM) and gave 95% pure Ab with a recovery yield of 90%. 

## 4. Modern Applications of Immunoglobulin Binding Ligands

**Figure 5:**
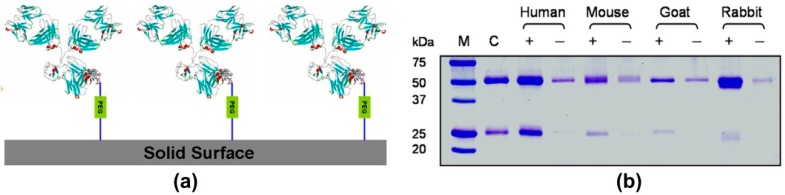
(**a**) Illustration of antibody binding on a solid surface using Fc-binding peptide Fc-III; (**b**) SDS-PAGE analysis of IgGs bound to Fc-III covered magnetic beads. Human IgG1 (*K*_d_ = 85 ± 5 nM), mouse IgG3 (*K*_d_ > 20 μM), goat IgG3 (*K*_d_ not given), and rabbit IgG (*K*_d_ = 305 ± 37 nM) were incubated with Fc-III functionalized (+) or unmodified (−) magnetic beads. After washing steps with PBS, the bound IgG were applied to a 10% SDS-polyacrylamide gel. Protein size marker (M) and 1 μg of human IgG (C) are indicated. Adapted from [70], with permission from © 2016 Elsevier.

Ongoing research in the development of Ab affinity ligands has resulted in the development of several novel applications. Fc-III was used by our group to immobilize Abs on the surface of several sensors with proper orientation [70]. In this approach, Fc-III was attached to the surface of gold chips, glass slides, and magnetic beads via a polar PEG linker (Figure 5). Next, the binding of various mammalian IgGs on the Fc-III decorated surfaces was analyzed using SPR. As expected, the antigen binding ability of the Abs that are captured using Fc-III was found to be superior compared to those with random orientation. 

**Figure 6:**
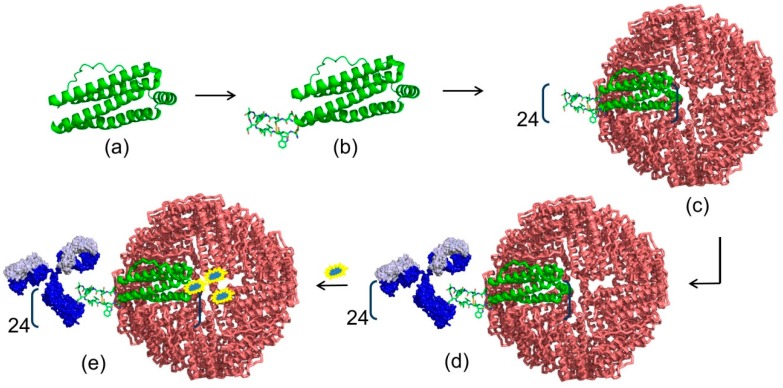
Illustration of the complexation of anti-Her2 antibody on a protein nanocage (ferritin) surface using the Fc-binding peptide Fc-III. (**a**) Ferritin monomer; (**b**) genetically engineered ferritin fusion monomer with Fc-III inserted in one of the loops; (**c**) self-assembled ferritin nanocage with 24 surface exposed Fc-III; (**d**) non-covalent binding of anti-Her2 antibody to the Fc-III functionalized protein nanocage; (**e**) covalent fluorescent labeling of the protein nanocage.

In addition we developed an Fc-III functionalized protein nanocage (bound to anti-Her2 Ab called trastuzumab or Herceptin) to specifically target breast cancer cells (Figure 6) [71,72]. Breast cancer cells are known to overexpress Her2 receptors on the cell surface. Protein nanocages readily self-assemble from (identical) subunits to form a stable protein nanocage. In this approach, ferritin (Ft), a 24-mer protein nanocage with a diameter of 13.2 nm was mutated to display the Fc-binding peptide Fc-III. To this end, the Ft monomer was genetically engineered to display Fc-III in one of the loops. After the monomers self-assembled to form the protein nanocage, Fc-III was clustered on the protein surface due to the highly symmetric nature, resulting in an enhanced avidity. Next trastuzumab was fluorescently labelled with Cy3 and localized on the protein surface through binding with Fc-III, followed by fluorescent labeling of the construct. 

**Figure 7:**
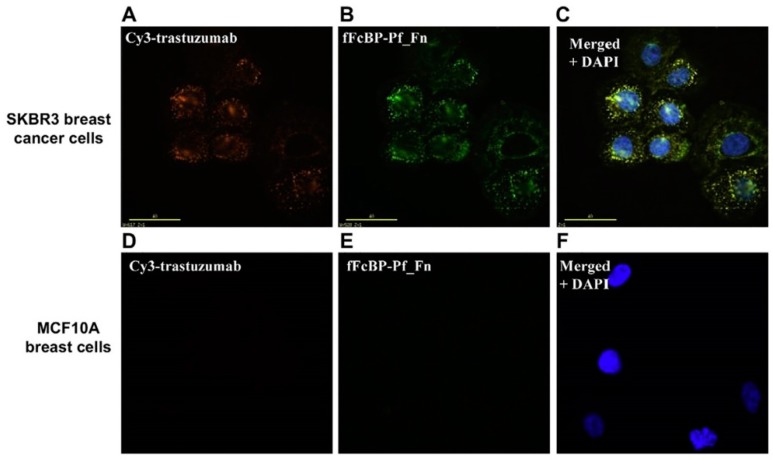
Fluorescent microscopic images of SKBR3 breast cancer cells (**A**–**C**) and MCF10A breast cells (**D**–**F**) treated with Cy3-trastuzumab/fFcBP-Pf_Fn complexes. Trastuzumab and FcBP-Pf_Fn were labeled with Cy3 (Cy3-trastuzumab) and fluorescein (fFcBP-Pf_Fn), respectively. Cy3-trastuzumab was simply mixed with fFcBP-Pf_Fn to form Cy3-trastuzumab/fFcBP-Pf_Fn complexes and subsequently incubated with SKBR3 breast cancer cells and MCF10A breast cells in the presence of excess amounts of rabbit serum. Cy3-trastuzumab and fFcBP-Pf_Fn are visualized as orange and green, respectively, by fluorescence microscopy. (For interpretation of the references to color in this figure legend, the reader is referred to the web version of this article.) Adapted from [71], with permission from © 2012 Elsevier.

SKBR3 breast cancer cells that overexpress Her receptors were treated with the functionalized nanocage (Figure 7). As a negative control, MCF10A breast cells that do not express Her receptors were used. Using fluorescent microscopy, it was clearly demonstrated that the Ab functionalized ferritin nanocage specifically targets the Her receptors on the SKBR3 breast cancer cells (MCF10A cells were not targeted). 

**Figure 8:**
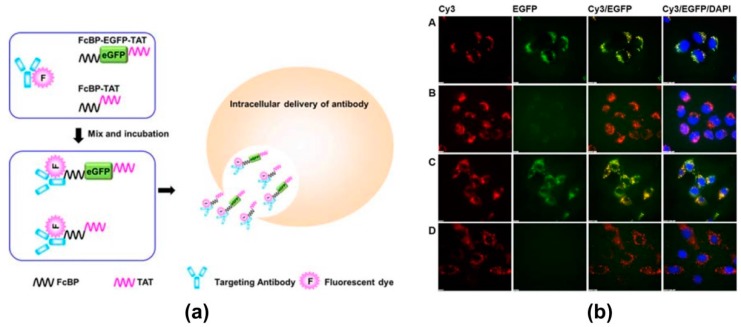
(**a**) Illustration of fluorescent labeled antibody delivery into live cells using the Fc-domain binding peptide (FcBP) Fc-III conjugated to the cell penetrating peptide Tat in addition to the green fluorescent protein; (**b**) Delivery of human IgG into live cells using the Fc-III/eGFP/Tat constructs. Cells were treated with Cy3 labelled human IgG (Cy3hIgG) in the presence of the Fc-III/eGFP/Tat or Fc-III/Tat. Cy3 (red): the fluorescence images of Cy3-hIgG (Ex/Em = 555/605 nm); EGFP: the fluorescence of eGFP the fluorescence (green) of eGFP (Ex/Em = 490/525 nm); Nucleus (blue) was stained with DAPI (Ex/Em = 350/455 nm); Cy3/eGFP: superimposition of Cy3-hIgG and FcBP-eGFP-Tat fluorescence images; Cy3/eGFP/DAPI: superimposition of Cy3-hIgG, FcBP-eGFP-Tat and DAPI fluorescence images. (A) HeLa cells treated with Cy3-hIgG and FcBP-eGFP-Tat; (B) HeLa cells treated with Cy3-hIgG and FcBP-Tat; (C) 3T3-L1 cells treated with Cy3-hIgG and FcBP-eGFP-Tat; (D) 3T3-L1 cells treated with Cy3-hIgG and FcBP-Tat. Adapted from [73], with permission from © 2015 Springer.

Recently our group developed an Ab transducer based on Fc-III, conjugated to the cell penetrating peptide Tat (Figure 8) [73]. In addition to the Fc-III/Tat conjugate, the fusions construct Fc-III/eGFP/Tat was prepared that includes the green fluorescent protein (GFP). Upon mixing of the two constructs in the cell growth media they readily targeted the Fc-domain of IgGs. Consequently, the targeted Abs translocated into the cytosol of live cells. The delivery of fluorescent labeled (Cy3, red) human IgG with the aid of the Fc-III/eGFP/Tat construct could be detected using fluorescent microscopy. 

**Figure 9:**
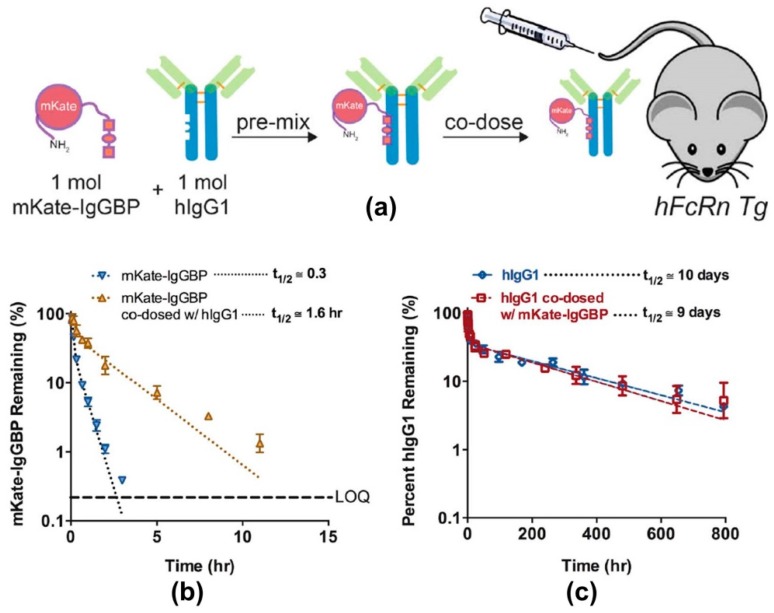
(**a**) Schematic of the co-administration scheme. In this experiment, human FcRn Tg mice were not pre-dosed with exogenous hIgG1. Instead mKate-IgGBP and hIgG1 were pre-mixed in a 1:1 molar ratio and co-injected via the tail vein; (**b**) clearance of mKate-IgGBP in hFcRn Tg mice dosed alone (blue triangles) or co-dosed at a 1:1 molar mixture with hIgG1 (yellow triangles). The mKate-IgGBP remaining (%) was calculated by normalizing the fluorescent emission at all time points to the maximum value observed in the first bleed 5 min after protein injection; (**c**) clearance of labeled human IgG1 in hFcRn Tg mice dosed as a single agent via the tail vein (blue triangles) compared to the clearance of labeled hIgG1 co-administered as a 1:1 molar mixture with mKate-IgGBP was measured to determine if bound mKate-IgGBP alters the eliminate profile of hIgG1 (red squares). The hIgG1 remaining (%) was calculated by normalizing the fluorescent emission at all time points to the maximum value observed in the first bleed 5 min after protein injection. Dashed lines in each panel represent the data fit to a 2-compartment PK model in Prism and the *β*-phase half-life shown in the figure was calculated as described in the Methods section. The data shown in each panel are the mean (*n* = 3 bleeds per time point) and error bars indicate standard deviation. Adapted from [74].

Sockolosky and coworker reported a substantial increase of the half-life of recombinant proteins that are fused to the Fc-binding protein Fc-III (Figure 9) [74]. The fusion of Fc-III to the C-terminus of a model far-red bright fluorescent protein, Katushka (mKate), specifically bind to human IgG (affinity: pH = 7.4–40 nM; pH = 6–20 nM). Interestingly, although Fc-III is known to interfere with FcRn binding (as discussed before, FcRn is involved in the endosomal salvation of IgG) the half-life of the Fc-III fusion protein showed a 75 fold increase (up to 8 h) in human FcRn transgenic mouse when the fusion protein and human IgG1 are mixed in a 1:1 ratio with respect to unmodified mKate.

**Scheme 1:**
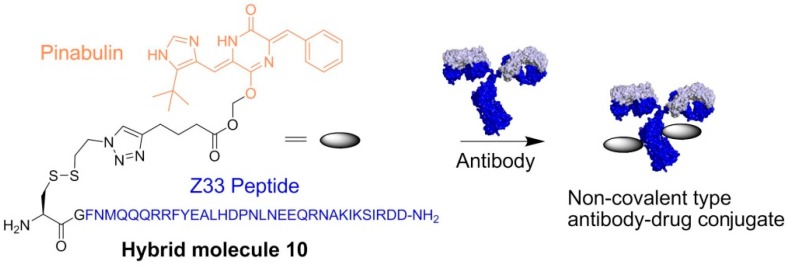
Preparation of non-covalent antibody—drug conjugate using Pinabulin (cytotoxic agent) and Fc-binding peptide Z33.

**Figure 10:**
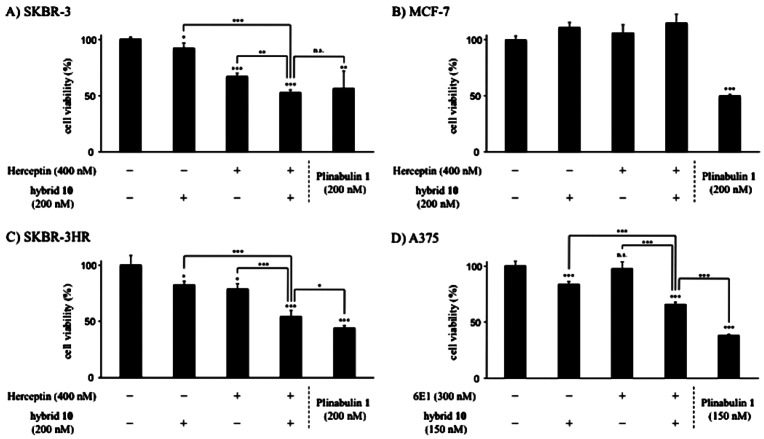
Cytotoxicity of NC-ADC, complex of hybrid 10 and Herceptin, against (**A**) SKBR-3 cells; (**B**) MCF-7 cells; and (**C**) SKBR-3HR cells; (**D**) cytotoxicity of noncovalent ADC, complex of hybrid 10 and 6E1 against A375 cell. n.s.: not significant, * *p* < 0.05, ** *p* < 0.01, *** *p* < 0.005. Data (*n* = 3) are shown as means ± SD. Adapted from [75], with permission from © 2016 American Chemical Society.

Muguruma and coworkers recently reported the development of a non-covalent antibody-drug conjugate (NC-ADC) based on the synthetic 33-mer Fc-binding peptide Z33 (Scheme 1) [75]. In their approach, the anticancer agent plinabulin was covalently linked to Z33 using Click chemistry. Next, high binding affinity of the cytotoxic conjugate to anti-Her2 Ab (Herceptin) and anti-CD71 Ab (6E1) was observed with a *K*_d_ = 46.6 ± 0.5 nM and 4.5 ± 0.56 μM, respectively. The prepared NC-ADC (with Herceptin) was evaluated against SKBR-3 cancer cells that overexpress Her-2 receptors versus MCF-7 cells that have low expression of Her-2 receptors (Figure 10). As expected, in cell based assays the NC-ADC shows a significant cytotoxicity in the presence of Herceptin towards SKBR-3 cells whereas the MCF-7 cells are not affected. In case of the NC-ADC with 6E1, similar results were obtained. 

## 5. Conclusions and Outlook

Immunoglobulin binding proteins have played a pivotal role in advancing antibody purification technologies. To this end, three key bacterial proteins (A, G, and L) have been extensively used and modified (both genetic and chemical) to enhance their properties for use in affinity purification chromatography of antibodies. The importance of these bacterial proteins has stimulated the search for synthetic immunoglobulin binding proteins. Especially, synthetic ligands that target the Fc-binding domain of antibodies are highly desirable because Fc-binding ligands do not interfere with the antigen binding ability of immunoglobulins. Several peptide based Fc-binding high affinity ligands were discovered over the past 20 years and used mainly in affinity chromatographic purifications of antibodies. However, as antibody technologies are becoming increasingly more important, Fc-binding ligands are finding broader applications. For instance, Fc-binding ligands have been used to bind antibodies on the surface of protein nanocages in targeted drug delivery approaches in anticancer therapeutics. In addition, Fc-binding ligands have been applied in the capturing of antibodies on solid surfaces in the development of biosensors as well as in the development of antibody-drug conjugates. Interestingly, Fc-binding ligands have been shown to inhibit FcRn binding. Because FcRn plays a crucial role in the long circulation times of antibodies, Fc-binding ligands holds high potential in the treatment of autoimmune diseases. The inhibition of FcRn binding can drastically influence the antibody half-life. 

## Figures and Tables

**Table 1:** General properties of the most used bacterial (fusion) proteins.

Protein	*K*_a_ *	Size	Target	Elution pH	References
Protein A	1.4 × 10^8^ M^−1^	42 kDa	Fc, Fab	3.5	[12,29]
Protein G	6.7 × 10^9^ M^−1^	30 kDa	Fc, Fab, ScFv, Dab	3.2	[28]
Protein L	5.69 × 10^7^ M^−1^	76 to 106 kDa	Fab, ScFv, Dab, and κ-light chain	2	[23]
Protein Z	5.0 × 10^8^ M^−1^	6.7 kDa	Fc	3.6	[24]
Protein LG	5.9 × 10^9^ M^−1^	50 kDa	Fc, κ-light chain	2	[25]
Protein LA	2.93 × 10^8^ M^−1^	60 kDa	Fc, κ-light chain	2	[26]
Protein AG	n.a.	47.3 kDa	Fc	3	[27]

* The depicted *K*_a_ values are towards IgG and may not be directly compared to each other because they were obtained at different conditions or by different methods. n.a.: Not available.

**Table 2:** A representable selection of high affinity (cyclic) peptides that selectively target immunoglobulin G.

Peptide	Binding Constant	Binding Capacity *	Elution pH	Remarks	Year (References)
**PAM**	*K*_d_ = 0.3 μM	n.a.	3 or 9	Dendrimer comb. library SpA mimic	1996 [45]
**d-PAM**	n.a.	50	3.5		2002 [46]
**d-PAM-Ф**	n.a.	10	4		2011 [47]
**TWKTSRISIF**	n.a.	n.a.	n.a.	Phage display library SpA mimic	1998 [48]
**FGRLVSSIRY**	n.a.	n.a.	n.a.		
**Fc-III (DeLano et al.)**	*K*_d_ = 16 nM			Phage display cyclic peptide library SpA mimic	2000 [42]
**Fc-III-(Sepharose)**		26.6 mg/μmol	3.5		2016 [49]
**FcBP-2**	*K*_d_ = 1.8 nM	n.a	n.a.	Bicyclic peptide	2006 [50]
**Fc-III-4C**	*K*_d_ = 2.5 nM	n.a.	3.5	Bicyclic peptide	2016[51]
**EPIHRSTLTALL**	n.a.	320 μg/g	n.a.	Phage display library SpA mimic	2001 [52]
**APAR**	*K*_d_ = 94 nM	9.1	n.a.	Comb. tetrapeptide library	2003 [53]
**FcRM**	*K*_d_ = 20 μM	n.a.	2.7	Synthetic cyclic peptide library, Fcγ-receptor mimic	2005 [54]
**HWRGWV**	*K*_d_ = 10 μM	28.4	4	Comb. library (one-bead-one-peptide) SpA mimic	2006 [55]
**HYFKFD**	*K*_d_ = 11 μM	27.0	n.a.		2009 [56]
**HFRRHL**	*K*_d_ = 26 μM	33.6	n.a.		2016 [57]
**HWCitGWV**	*K*_d_ = 108 μM	72	n.a.		
**D_2_AAG**	*K*_a_ = 7.9 × 10^5^ M^−1^	36.2	3.6	Comb. library SpA mimic	2012 [58]
**DAAG**	*K*_a_ = 2.6 × 10^6^ M^−1^	49.6	3.6		
**cyclo[(Nα-Ac)S(A)-RWHYFK-Lact-E]**	n.a.	n.a.	3.5	Cyclic peptide	2013 [59]
**cyclo[(Nα-Ac)–Dap(A)-RWHYFK-Lact-E]**	n.a.	n.a.	3.5		
**cyclo[Link-M-WFRHYK]**	*K*_d_ = 7.6 μM	19.7	4.0	mRNA display library SpA mimic	2013 [60]
**NKFRGKYK**	*K*_a_ = 8.6 × 10^6^ M^−1^	DBC-4.9	4.0	Spot peptide array Fcγ mimic	2013 [61]
**NARKFYKG**	*K*_a_ = 6.5 × 10^6^ M^−1^	DBC-5.0	4.0		
**FYWHCLDE(1)**	*K*_d_ = 1.5 μM	104.2	6.0	Biomimetic design strategy	2014 [62]
**FYCHWALE(2)**	*K*_d_ = 6.1 μM	87.6	6.0	Fc-binder (SpA mimic)	2014 [63]
**FYCHTIDE(3)**	*K*_d_ = 5.7 μM	63.7	6.0		2014 [64]
**Dual 1/3 (2:1)**	*K*_d_ = 0.69 μM	137.9	6.0	Dual affinity ligand	2014 [65]
**RRGW**	*K*_d_ = 0.5 nM	n.a.	n.a.	Computer design strategy Fc-binder	2014 [66]
**KHRFNKD**	*K*_d_ = 20 nM	n.a.	n.a.	Phage-display library SpA mimic	2015 [67]

* Maximal binding capacity (q_m_) in mg/mL. DBC: Dynamic binding capacity. The depicted values are towards IgG and may not be directly compared to each other because they were obtained at different conditions or by different methods. n.a.: Not available.

**Table 3:** Affinities of various ligands that target the Fc-domain of IgG. IC_50_ values are from competition BIAcore assays using Protein A with standard deviations from at least three independent experiments. *K*_i_ values were determined by the method described by DeLano et al. [42]. Adapted from [50], with permission from © 2006 ACS publications.

Ligand	IC_50_ (nM)	*K*_i_ (nM)
Fc-III, pH = 7.4	460 ± 74	215
Fc-III, pH = 6.0	280 ± 41	41
Fc-III, reduced	>10^6^	n.d.
FcBP-1, pH = 7.4	64,800 ± 4400	14,200
FcBP-1, pH = 6.0	395,000 ± 7100	71,000
FcBP-2, pH = 7.4	32 ± 2	0.4
FcBP-2, pH = 6.0	35 ± 2	2.0
FcBP-2, reduced, pH = 7.4	550 ± 50	n.d.

n.d.: Not determined.
